# The relationship between job performance and perceived organizational support in faculty members at Chinese universities: a questionnaire survey

**DOI:** 10.1186/1472-6920-14-50

**Published:** 2014-03-13

**Authors:** Xin Guan, Tao Sun, Yan Hou, Liang Zhao, Yi-Ze Luan, Li-Hua Fan

**Affiliations:** 1 Department of Health Service Management, School of Public Health, Harbin Medical University, Harbin 150081, China; 2 Department of Epidemiology and Biostatistics, School of Public Health, Harbin Medical University, Harbin, China

**Keywords:** Job performance, Perceived organizational support, Chinese university, Faculty members

## Abstract

**Background:**

Although several studies have been conducted to investigate the relationship between perceived organizational support (POS) and job performance (JP), it remains unclear whether this relationship is appropriate for faculty members at Chinese universities. The objectives of this study were to (a) examine the correlation between POS andJP; (b) identify the predictors of POS, including demographic and organizational characteristics among faculty members at a Chinese university; (c) investigate the influence of mediating factors between POS and JP; and (d) compare the findings of this study with related studies.

**Methods:**

A cross-sectional questionnaire survey was used in this study. The questionnaire was administered to 700 faculty members who were randomly selected from all faculty members at six universities. A total of 581 questionnaires were obtained. A statistical model for JP was developed based on the literature review.

**Results:**

The analysis results indicated that the relationship between POS and JP was mediated by job satisfaction (JS), positive affectivity (PA), and affective commitment (AC). In addition, procedural and distributive justice contribute to POS.

**Conclusions:**

The study concludes that the relationship between POS and JP is mediated by JS, PA, and AC and is influenced by POS. These results can provide evidence for university administrators to improve POS and increase the JP of faculty members at universities.

## Background

It is generally believed that universities perform well in the areas of teaching and research in terms of discovering and developing talent for the development of science and technology [1]. Each university conducts an annual review and an evaluation of faculty job performance (JP). The items for evaluation include instructional responsibility, intellectual contribution, professional service, collegiality, and professionalism [2]. These areas are used for the determination of salary increases, qualification for promotion and tenure, reappointment of non-tenured faculty, and faculty awards in China [3]. So far, some studies have been performed to investigate the relationship between JP and pressure [4]. However, the relationship between perceived organizational support (POS) and JP among university faculties has not been investigated.

According to organizational support theory, POS reflects the degree to which employees believe that their work organization values their contribution and cares about their well-being [5,6]. POS could produce a felt obligation to care about the organization’s welfare and to help the organization achieve its goal [7]. Meanwhile, POS should fulfill socioemotional needs by incorporating organizational membership and role status into their social identity and strengthen employees’ beliefs that organization rewards increased performance [6]. Employers want employees to be dedicated and loyal to their work. If employers provide a high level of support to their employees, based on the norm of reciprocity, employees are likely to emotionally commit to their organizations with a low likelihood of turnover and a high level of job performance [8-10]. In a meta-analysis of 70 studies, Rhoades et al. demonstrated that employees’ POS could increase JP [6]. However, some previous studies have presented inconsistent results. Stamper et al. reported that POS was unrelated to task performance among salespeople [11]. Moreover, previous evidence has suggested that POS mediates numerous types of organizational experience variables and may not directly influence job performance [6,7,11]. Therefore, it is unknown whether POS is directly correlated with JP or is mediated by other factors for university faculty members.In our study, we investigate the relationships between antecedents (two dimensions of organizational justice and demographic characteristics) and POS and between POS and JP. Specifically, we consider the mediating roles of job satisfaction (JS), positive affectivity (PA), and affective commitment (AC) in the association between POS and JP in faculty members from a Chinese university.

### Hypothesis model

**Figure 1:**
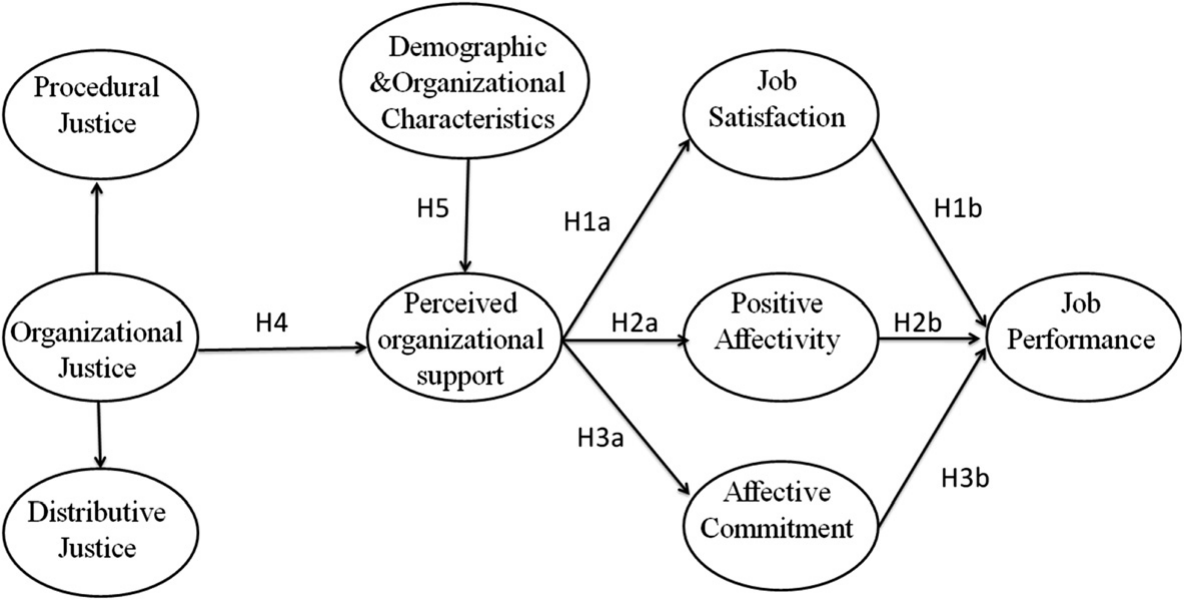
Hypothetical model of the relationships between antecedents and POS, the direct association between POS and JP, and the mediating effect of job satisfaction, positive affectivity, and affective commitment in the association between POS and JP among faculty members from a Chinese university.

Figure 1 outlines the hypothetical model of the present study, which will be described in detail below.

In our model, the core feature is POS. Based on the studies about the psychological processes underlying consequences of POS from Rhoades and Eisenberger [6], it is found that POS should produce a felt obligation to care about the organization’s welfare and to help the organization reach its objectives. Meanwhile, the caring, approval, and respect connoted, by POS should fulfill socioemotional needs, helping workers to incorporate organizational membership and role status into their social identity. Besides, POS should strengthen employees’ beliefs that the organization recognizes and rewards increased performance. These processes should have favorable outcomes both for employees and for the organization.

### Job satisfaction as a mediator of the relationship between POS and JP

Job satisfaction reflects an individual’s level of contentment with his or her job. During a social exchange, a person identifies the amount of input gained from a relationship compared to the output as well as the amount of effort the other person puts forth [12]. An increase in the help delivered to a recipient has been found to increase the aid returned and the liking for the donor [13]. According to organizational support theory, POS meets the need for social emotion, but praise and approval are still needed [5]. Job satisfaction refers to employees' attitude toward their job [14]. POS should can meet socioemotional needs and increase performance-reward expectancies, or signal the availability of aid, which could contribute to overall job satisfaction. Therefore, we assume that POS is positively related to job satisfaction.

Hypothesis 1a: POS is positively related to employees’ job satisfaction.

The Hawthorne studies indicate that the relationship between workplace attitudes and productivity has been a venerable research tradition [15]. Previous studies have presumed a relationship between attitude and behavior [16-19]. Due to human relations movement, satisfaction follows from the rewards produced by performance. Eagly and Chaiken (1993) concluded that people who evaluate an attitude object favorably (that is, high level of job satisfaction) tend to engage in behaviors that foster or support it, and people who evaluate an attitude object unfavorably (that is, low level of job satisfaction) tend to engage in behaviors that hinder or oppose it [20]. Following this logic, the attitude towards a job should influence behavior in the workplace, of which job performance is the central part [18]. Additionally, we are interested in the possible mediating effect of employees’ sense of satisfaction in terms of POS and performance [6]. Therefore, we propose the following hypothesis:

Hypothesis 1b: Job satisfaction is positively related to employees’ performance and partially mediates the relationship between POS and performance.

### Positive affectivity as a mediator of the relationship between POS and JP

Positive affectivity is a characteristic that describes how animals and humans experience positive emotions and interact with others and with their surroundings [21]. Watson, Clark, and Tellegen (1988) defined positive affectivity as involving feelings of enthusiasm, excitement, confidence, and alertness [22]. Positive affectivity, which is also referred to as positive mood, can be enhanced by events at work that indicate an employee’s competence, worth, or achievement [23]. Meyer and Allen (1991) suggested that emotional attachment was enhanced by work experiences contributing to employee comfort and perceived competence. POS might contribute to such experiences, fostering positive affectivity [7]. George and Brief (1992) proposed that events at work reflecting an employee's competence, worth, or achievement would enhance positive affectivity [23]. POS may contribute to positive affectivity by conveying an organization’s positive valuation of an employee’s work and care for the employee’s well-being [7]. Therefore, we proposed the following hypothesis:

Hypothesis 2a: POS is positively related to employees’ positive affectivity.

George and Brief (1992) noted that positive affectivity was a pivotal construct in the model and posited that it was the direct precursor of organizational spontaneity. It includes helping co-workers, protecting the organization, and making constructive suggestions, which are performance-related behaviors [23]. Performance is a function of ability and motivation [24-26]. The existing research on positive affectivity has suggested that positive feeling states influence the ability component of task performance in a variety of ways [25,27,28]. Kaplan et al. (2009) proved a positive relationship between PA and job performance [29]. They concluded that higher PA is associated with greater expectancy and optimism, in turn, it should foster different behaviors beneficial to performance. For instance, those high in PA may select more demanding goals [30], demonstrate greater determination, engage in effective problem-solving strategies [31], and utilize more efficacious coping strategies [32]. Thus, whereas other workers may show less initiative or fail to persevere, the positive expectations held by higher PA individuals ultimately should result in their selecting and completing challenging work tasks [29]. Due to other mediating variables, such as job satisfaction and affective commitment, we believe that positive affectivity partially mediates the relationship between POS and employees’ job performance.

Hypothesis 2b: Positive affectivity is positively related to employees’ performance and partially mediates the relationship between POS and JP.

### Affective commitment as a mediator of the relationship between POS and JP

Affective commitment (AC) is defined as an employee’s positive emotional attachment to an organization and is a component of organizational commitment [33]. Eisenberger et al. proposed that POS increases affective commitment partly by making employees feel obligated to care about and aid their workplace [7]. Specifically, based on the reciprocity norm, POS should create a perceived obligation to care about the organization’s welfare and to help the organization achieve its goals. Employees can fulfill this indebtedness through greater AC [7]. Moreover, Rhoades et al. concluded that there is an unidirectional association between POS and AC based on repeated measurement data.

Meyer and Allen (1991) considered a positive emotional attachment by employees to their work organization as a distinct type of organizational commitment [33]. Tsui et al. (1997) suggested that caring and positive regard for employees from organization act to enhance affective commitment via the reciprocity norm [34]. Organizational support theory supposes that POS contributes to affective commitment by creating a felt obligation to care about the organization and meet the organization's objectives [7]. Consequently, POS is positively related to changes in AC over time, providing evidence that POS contributes to AC.

Hypothesis 3a: POS is positively related to employees’ affective commitment.

From a social exchange perspective, commitment is likely to be the central factor in the reciprocity between employees and their organizations [5,35]. Because affective commitment has a positive effect on employees, an impressive number of studies have linked affective commitment to workplace outcomes such as task performance, promotion capacity, and organizational citizenship behavior [36-40]. Meyer and Allen (1991) argued that employees want to stay in organizations that provide them with positive work experiences since they highly value these experiences and expect them to continue. Moreover, they are likely to contribute to organizational effectiveness by maintaining equity in their relationship with the organization. For employees who perform at a high level of proficiency may consequently develop a more positive attitude (affective commitment) toward the organization. Such an attitude may ensure the continuation of a high level of performance in the future [33]. Additionally, employees who perceive that their organization cares about and values them seem to develop stronger affective commitment to their organization. Choi (2006) found affective commitment to be a partial mediator of the relationship between POS and contextual performance [41]. Takeuchi et al.’s longitudinal survey also demonstrated a positive correlation between POS for a current assignment and job performance, which is also affected by affective commitment [35]. In the present study, given that several different variables may act as mediators between POS and JP, we consider the possibility that employees’ affective commitment may partially mediate the relationship between POS and JP.

Hypothesis 3b: Affective commitment is positively related to employees’ performance and partially mediates the relationship between POS and JP.

### Antecedent variables of POS

Based on organizational support theory, POS is influenced by various aspects of an employee’s treatment by his or her organization. Specifically, these aspects include the organization’s desire to pay a fair salary and to make the employee’s job meaningful and interesting [5]. In the present study, we propose that organizational justice (especially procedural justice and distributive justice) and personal and organizational characteristics contribute to perceived organizational support.

As defined by Greenberg (1987), organizational justice or fairness is related to how an employee judges the behavior of an organization and the resulting attitude and behavior. There are three main proposed components of organizational justice: distributive justice, procedural justice, and interactional justice [42]. Distributive justice is conceptualized as the fairness associated with decision outcomes and the distribution of resources [43]. The distributed outcomes or resources may be tangible (e.g., pay) or intangible (e.g., praise). According to organizational support theory, favorable opportunities for rewards communicate a positive valuation of employees’ contributions and thus contribute to POS [5,6,44]. Procedural justice is defined as the fairness of the process that leads to outcomes. When individuals feel that they have a voice in the process or that the process involves characteristics such as consistency, accuracy, ethicality, and a lack of bias, procedural justice is enhanced [45]. Procedural justice is used to determine the distribution of resources among employees [46]. Shore and Shore (1995) suggested that repeated instances of fairness in decisions concerning resource distribution should have a strong cumulative effect on POS by indicating a concern for employees’ welfare [47]. Rhoades et al. reported that more than 70 studies suggest that basic antecedents of POS include fair organizational procedures, supervisor support, and favorable rewards and job conditions (which are the same as distributive justice) [6].

Hypothesis 4: Organizational justice is positively related to employees’ POS. Specifically, procedural and distributive justice contribute to POS.

The demographic characteristics of employees are often used as control variables to rule out alternative explanations for the relationship between two hypothesized variables. These characteristics may include age, which is related to remaining working time [48]; gender, which is related to job performance and turnover [49]; work experience, which is related to job performance and intent to quit [50]; and organizational tenure, which is related to POS and intent to quit [50]. The present study used personal demographic characteristics (e.g., age, gender, teaching tenure) and organizational characteristics (e.g., subject type and school type) as control variables.

Hypothesis 5: Demographic and organizational characteristics are positively related to employees’ POS.

## Methods

### Sample and procedure

We randomly selected 700 faculty members from six public universities for a questionnaire survey in Heilongjiang Province of China. Of the 700 distributed questionnaires, 581 were returned, for an 83.0% effective rate. The characteristics of all participants are summarized in Additional file 1. Of these 581 participants, 220 are male with an average income of RMB3726.6 per month, and 361 are female with an average income of RMB3644.3 per month (*p* = 0.42). The proportion of each age was 22.6%, 63.7%, 11.3%, and 2.4%, respectively.

### Measures

Because all scales were translated from English and translation differences between the English and Chinese versions are inevitable, confirmatory factor analyses (CFA) was conducted to validate the construction of all scales. We followed a procedure to assess the covariance matrix of all items presenting a unique scale and then established a one-factor CFA model to identify whether the model was well fitted [51]. All scales in this questionnaire were on a seven-point Likert-type scale format (1 = disagree extremely to 7 = agree extremely).

### Perceived organizational support scale

The items on the POS scale were those with high factor loadings from the questionnaires developed by Eisenberger et al. in 1986. In 2002, Eisenberger re-confirmed the adaptability of these items using meta-analysis methods [5,6]. Sample items were presented, such as, ‘When I am in trouble, employers will help me’, ‘Employers care about my welfare’, and ‘Employers are very concerned about me’.

### Job performance scale

The five-item job performance scale was developed by Williams and Anderson in 1991 and was initially used to measure individuals’ overall performance level, task completion, and competency [52]. This job performance scale was based on participants’ self-reports. Sample items include ‘I can competently complete assigned work’, ‘I can perform the duties of my job description’, and ‘I never neglect my job responsibilities’.

### Organizational justice scale

Items related to organizational justice were separated into two dimensions, procedural justice and distributive justice. The present study measured these two types of justice using scales developed by Erdogan, Liden, and Kraimer [53]. There are five items on procedural justice scale. Sample items include ‘My superior makes fair work-related decisions’, ‘When making a work-related decision, my superior does his best to collect relevant, accurate, and complete information’, and ‘When I request, my superior clarifies his decisions and provides additional information’. The distributive justice scale also has five items, including ‘All work-related decisions are executed by all of my work-related peers’, ‘Overall, I receive a fair and reasonable reward’, and ‘My duties are appropriately fair and reasonable’.

### Job satisfaction scale

The job satisfaction scale aims to assess whether an individual is satisfied with his or her job. This scale, developed by Smith et al. [54], is a five-item scale. Sample items include ‘I am quite satisfied with my present job’, ‘Most days, I am enthusiastic about my work’, and ‘I find it enjoyable to do my work’.

### Affective commitment scale

Affective commitment to the workplace was measured using an 18-item organizational scale developed by Meyer and Allen [33]. We selected five items to assess the affective commitment dimension, including ‘I would be happy to work at my school until I retire’, ‘I feel like part of a family at my school’, and ‘I feel a strong sense of belonging to my school’.

### Positive affectivity scale

This scale indicates whether a participant has a positive mood in his or her daily work. Using the proactive personality scale developed by Seibert et al. [55], ten items with high factor loadings were selected. Sample items include ‘I play one of the most important roles wherever I am’, ‘I would do my best to overcome without interest’, and ‘I continue to look for new and better ways to complete my work’.

### Analytic strategy

First, we examined the construct validity of POS, JP, JS, PA, PC, OJ, and procedural and distributive justice. To illustrate that all items evaluating these variables were unidimensional, we followed Shore and Tetrick’s (1991) procedure to assess the covariance matrix of the items for each measure against a one-factor model [51].

Then, we performed a mediational analysis to determine whether there were significant mediating effects of job satisfaction, positive affectivity, and positive commitment between POS and job performance. Because there are three mediators in the model, we followed Preacher and Hayes’s (2008) product-of-coefficients approach to assess the mediating effects in a multiple mediator model [56]. We computed both the specific and total indirect effects of mediators. To compute the specific indirect effect, we first tested whether either of the constituent paths for a hypothesized indirect effect (that is, A_i_ and B_i,_ where i equals job satisfaction, positive affectivity, or positive commitment) through the variable Mi were not different from zero. If both paths are not zero, the indirect effect of that specific mediator is significant and equals Ai*Bi. To compute the total indirect effect, we conducted the Sobel test (see Preacher and Hayes 2008) to provide the significance test for a multiple mediated effect. Estimates of all path coefficients were calculated using an ordinary least squares (OLS) regression. Because the assumption of normality of the sampling distribution of the indirect effect may be violated, we followed Preacher’s recommendation to use a bootstrap technique to correct the bias and to increase the confidence interval of estimates of the indirect effect [56].

Hierarchical regression analysis was used to test whether organizational justice contributed to POS. In the first step, demographic and organizational characteristics were used as covariates in the predictor equation. Then, we included the independent variables of procedural and distributive justice in the equation to test the direct effect. The direct effect was determined using the amount of additional explained variance (delta R^2^) after entering organizational justice.

The CFA analysis was performed in LISREL 8.80, and other analyses were performed using SAS (version 9.2, SAS institute, Cary, NC).

## Results

Prior to examining the relations among POS, PJ, DJ, JS, PA, AC, and performance, we submitted each subscale to confirmatory factor analysis to establish its unidimensionality. In addition, we also conducted CFA using all itmes and 7 subscales as latent variables to test the unidimensionality of the whole questionnaire. The results of the CFAs are shown in Table 1. A single-latent-factor model was found to adequately account for the covariance matrices among all of the scales.

**Table 1:** Fit Results of Tests of Unidimensionality for Procedural Justice (PJ), Distributive Justice (DJ), Perceived Organizational Support (POS), Job Satisfaction (JS), Positive Affectivity (PA), Affective Commitment (AC), and Performance

**Scale**	**No. of items**	**χ**^**2**^	**df**	**Fit indices**			
				**GFI**	**CFI**	**NFI**	**SRMR**
Procedural Justice (PJ)	5	132.83	5	.92	.97	.96	.03
Distributive Justice (DJ)	5	354.64	5	.80	.88	.88	.07
POS	8	333.22	20	.87	.96	.96	.04
Job Satisfaction (JS)	5	54.45	5	.96	.94	.94	.06
Positive Affectivity (PA)	10	661.70	35	.81	.92	.92	.08
Affective Commitment (AC)	6	87.55	9	.95	.98	.98	.03
Performance	5	76.69	5	.95	.98	.98	.02
The whole scales	44	2038.33	805	.86	.95	.90	.10

Note. All chi-squared values were significant, p < .001. GFI = goodness of fit index; CFI = comparative fit index; NFI = normed fit index; SRMR = standardized root mean square residual.

The parameters for the goodness of fit of the estimated models included the ratio of chi-squared to degrees of freedom, the goodness-of-fit index (GFI), the comparative fit index (CFI), the normed fit index (NFI), and the standardized root-mean-square residual (SRMR). The CFI is an incremental measure that is directly based on the non-centrality measure. The NFI was the first measure of fit proposed in the literature [57]. GFI, CFI, and NFI values above 0.9 suggest a reasonable fit. The SRMR is an absolute measure of fit and is defined as the standardized difference between the observed correlation and the predicted correlation. A value less than 0.08 is generally considered a good fit [58]. From the analysis results in Table 1, we can easily conclude that the seven designed scales had good unidimensionality.

After we obtained evidence of the unidimensionality of the designed scales, we used a single indicator to represent one scale. To enhance the representativeness of a specific scale, we reduced the number of items in that scale using the following procedure [59]. First, we fit separate one-factor solutions for each of these scales. Then, we created aggregate items by averaging items with high and low loadings. For example, for a five-item scale, we created the first aggregate item by averaging the items with the highest and the lowest loadings, the second by averaging the items with the second-highest and the second-lowest loadings, and the third by retaining the items with the middle loadings. Then, we obtained the composite indicator of that scale by averaging the remaining items. Table 2 presents the means, standard deviations, zero-order correlations, and estimates of internal consistency (Cronbach’s alpha coefficient) for all variables measured in the present study. The respondents reported a comparatively high level of correlation between POS, JS, and AC and a moderate level of correlation between POS and PA and between POS and JP. These significant correlations were consistent with hypotheses 1a, 2a, and 3a. Regarding hypotheses 1b, 2b and 3b, JS, PA, and AC were positively correlated with performance. Therefore, we can conclude that the mediators JS, PA, and AC may mediate the effect of POS on performance. In Table 2, we also see that procedural justice (PJ) and distributive justice (DJ) were both significantly and positively correlated with POS, which is consistent with hypothesis 4. To estimate the reliability of the designed scales, we used the Cronbach’s alpha coefficient [60]. Following the rule of thumb [61], we obtained excellent internal consistency for the scales of PJ, DJ, POS, PA, AC, and performance. The internal consistency of the job satisfaction scale was poor but acceptable.

**Table 2:** Descriptive statistics and zero-order correlations

	**Mean**	**SD**	**1**	**2**	**3**	**4**	**5**	**6**	**7**
1 Procedural Justice (PJ)	4.92	1.57	(.95)						
2 Distributive Justice (DJ)	4.56	1.52	.73	(.92)					
3 POS	4.08	1.44	.66	.67	(.94)				
4 Job Satisfaction (JS)	4.63	1.53	.56	.66	.56	(.56)			
5 Positive Affectivity (PA)	4.94	1.11	.37	.34	.29	.37	(.91)		
6 Affective Commitment (AC)	4.76	1.33	.57	.60	.56	.70	.42	(.91)	
7 Performance	5.70	1.30	.39	.33	.28	.41	.50	.47	(.97)

Note. correlation coefficients were significant, *p*<.001; Cronbach’s alpha coefficients are in parentheses.

POS influenced job performance indirectly via job satisfaction, positive affectivity, and affective commitment. This suggests a mediating effect model. Specifically, in the present model, the effect of POS on job performance is a direct effect, and there are indirect effects of POS on job performance via the three mediators of job satisfaction, positive affectivity, and positive commitment. We first examined the direct effect by constructing a model only with the independent variable POS and the dependent variable job performance and labeled the path coefficient of the independent variable as C. We then examined the relationship between the independent variable POS and the mediators, whose path coefficients are labeled A. Finally, we examined the relationship between the mediators and the dependent variable of job performance when controlling for the independent variable; the path coefficients of the mediators are labeled B, and the independent variable is labeled C’.

There are three sets of relationships involving mediators: (1) POS—JS—JP; (2) POS—PA—JP; and (3) POS—AC—JP. The results are shown in Table 3. The path coefficients for A and B were both significant. Similarly, the path coefficients for all steps involving POS, PA, and JP were significant, as were the steps involving POS, affective commitment, and performance. Thus, hypotheses 1a, 1b, 2a, 2b, 3a, and 3b were validated.

**Table 3:** Path coefficients of mediators and fit statistics

**Path**	**Label**	**Coefficient**	**SE**	**t value**	**R**^**2**^
POS—JS	A	0.60	0.04	16.33***	
JS—JP	B	0.09	0.04	2.16*	
POS—PA	A	0.22	0.03	7.22***	
PA—JP	B	0.42	0.04	9.44***	
POS—AC	A	0.52	0.03	16.28***	
AC—JP	B	0.25	0.05	5.14***	
POS—JP	C	0.25	0.04	6.99***	
POS—M—JP	C’	−0.03	0.04	-.74	
Model					0.33

Note. POS=Perceived Organizational Support; JP=Job Performance; JS=Job Satisfaction; PA=Positive Affectivity; AC=Affective Commitment; A, path coefficient for independent variable on mediators; B, path coefficient for mediators on dependent variables; C, path coefficient for independent variable on dependent variable; C’, path coefficient for independent variable on dependent variable when controlling mediators. *p<.05 ***p<.001.

In Table 3, we can see that the effect of POS (labeled C) positively predicts JP. When mediators were added, the effect of POS (labeled C’) was not statistically significant. We can compute the total indirect effect through the difference between these two regression parameters (C-C’). Mackinnon et al. (1995) proved the equivalence of this method and the approach of multiplying the two regression parameters (A*B) involved above [62]. In this study, the POS effect C’ is not different from zero when the mediators are included in the model, which indicates that the program effect is entirely mediated by the mediating variable. This is consistent with the hypothesis mentioned above that POS may not directly predict JP.

We also conducted a multiple mediation model including three mediators. Table 4 presents the analysis results. The specific indirect effects are a1*b1 = .05 (through JS), a2*b2 = .09 (through PA), and a3*b3 = .13 (through AC). Of the potential mediators examined, we can conclude that these three mediators are all significant, as is the total indirect effect. This strongly proves that our hypotheses 1a, 1b, 2a, 2b, 3a, and 3b are supported.

**Table 4:** Mediation of the effect of perceived organizational support on performance through job satisfaction, positive affectivity, and affective commitment

		**Product of coefficient**		**BCa 95% CI**	
**Mediator**	**Point estimate**	**SE**	**Z**	**Lower**	**Upper**
Job satisfaction	.05	.02	2.14*	.0016	.1122
Positive affectivity	.09	.02	5.80***	.0601	.1339
Affective commitment	.13	.03	4.92***	.0674	.2057
Total	.28	.03	9.28***	.2130	.3566

Note. bootstrap sample = 1000; BCa = bias corrected and accelerated *p<.05; ***p<.001.

Because the assumption of normality of the sampling distribution of the total and specific indirect effects is questionable, we bootstrapped the indirect effects using the SAS version of the macro [56]. The estimates and accelerated confidence intervals (CIs) are shown in Table 4. The bootstrap estimates presented here were based on 1000 samples. The results indicated that JS, PA, and AC mediate the effect of POS on performance. As shown in Table 3, the total and direct effects of POS on performance are 0.25 (p < .001) and −0.03 (p > .05), respectively. The difference between the total and direct effects is the total indirect effect through the three mediators, with a point estimate of 0.28 and a 95% CI of 0.2130 to 0.3566 (i.e., we can claim that the difference between the total and the direct effect of POS on performance is not different from zero).

The hierarchical multiple regression is used to test the hypotheses more thoroughly. In step 1, the multivariate model involves covariates, demographic, and organizational characteristics. In step 2, organizational justice, procedural justice, and distributive justice were included in the analysis model. The results of the hierarchical regressions are shown in Table 5. Although model 1 (with only independent variables for demographic and organizational characteristics) was significant, no specific demographic or organizational covariate was significant. Therefore, we concluded that demographic and organizational characteristics cannot predict POS, and the hypothesis was not supported. In model 2 (with independent variables for procedural justice, distributive justice, and all covariates), all predictors yielded 53% of the variation, of which 49% of the variation was produced by procedural and distributive justice. Because the standardized regression coefficients of procedural and distributive justice are positive, these two dimensions of organizational justice can positively predict POS. The results suggest that the hypothesis is supported.

**Table 5:** Results of hierarchical multiple regression analysis regarding the contribution of demographic and organizational characteristics, procedural justice, and distributive justice to the explanation of variance of POS

**Predictor**	**beta**_**s**_^**a**^	***t *** **value**	**R**^**2**^	**R**^**2 **^**change**
	The first step			
Demographic characteristics				
Sex	.01	.17		
Age	-.11	−1.83		
Educational level	-.05	−1.00		
Tenure	-.09	−1.51		
Job title	.00	.03		
Income level	.00	-.03		
Total		1.87**	.04	
Organizational characteristics				
School level	−0.10	.25		
Subject level	−0.12	−1.81		
Total		1.30	.01	
Total of covariates		1.72**	.04	.04
	The second step			
Organizational justice				
Procedural justice	.41	9.57***		
Distributive justice	.35	8.23***		
Total		17.29***	.49	.49
Total of all predictors		8.04***	.53	

Note. a = standardized regression coefficient. **p<.01 ***p<.001.

## Discussion

Diverse samples of employees from different levels of universities in China are used in this study. The analysis results indicate that POS is positively related to JS, PA, and AC, and these three mediators are positively associated with JP. These findings are consistent with organizational support theory. Eisenberger et al. (2001) found that the relationship between POS and AC is partly mediated by employees’ perceived obligation to care about the welfare of the organization and to help the organization achieve its goals. Eisenberger et al. (1997) reported that favorable work experiences is associated with POS, and employees believe these to be under the organization’s control. In this study, we present the mediating effect of proposed variables. We firstly assume that without any mediating variables, POS would positively predict JP and then with these variables the effect of POS is not statistically significant, which suggests that the relationship between POS and JP is entirely mediated by JS, PA, and AC. Employees who perceive that their organization cares about and values them seem to develop stronger affective commitment to their organization. Choi found that AC is a partial mediator of the relationship between POS and contextual performance [41]. Takeuchi et al.’s longitudinal survey also demonstrated a positive correlation between current assignment POS and JP, which is affected by AC [35]. The results suggest that organizations must improve JS, PA, and AC for employees by taking various measures to increase the JP of university faculty members.

We also find that procedural and distributive justice are positively related to employees’ POS. This finding is consistent with organizational support theory, which suggests that favorable opportunities for rewards communicate a positive valuation of employees’ contributions and contribute to POS [5,6]. The hierarchical multiple regression analysis results revealed that procedural and distributive justice explain almost half of the variation of POS, and these two dimensions of organizational justice can positively predict POS.

The purpose of this study was to investigate how to achieve the organizational effectiveness, which is a major concern of organizational theorists and practitioners in faculty staff in Chinese university and could help better understand the relationships between POS, JS and JP in China. One objective is to examine the extent to which POS is associated with JP. Another is to investigate the relationships between JS and JP.

The approach that we used in this study gives us a more precise understanding of the relationship between POS and JP. The effects of mediators in this relationship indicated that POS is positively related to JS, PA, and AC, and these three mediators are positively associated with JP, which partially explain the long confusion in past regarding the relationship between POS and JP. This highlights the importance of choosing mediators and criteria in terms of compatibility, both conceptually and empirically.

Since once medical educators are hired by universities in China and they could work until retire without any pressure about the work. Therefore, some of them might have no motivation to develop their instructional responsibility, intellectual contribution, professional service, collegiality, and professionalism. For this reason, we must create an effective system to raise faculty members’ job performance. Our results provide the administrator information on how to improve the job performance of faculty members in university in China. According o our results, administrators could increase their JP through POS, and POS could be improved by enhancing job satisfaction, positive affectivity, affective commitment, procedural and distributive justice. It is generally suggested that administers should seek to increase the level of support given to these staff by universities. By implementing policies and work processes sending information to employees that the organization cares about their well-being and highly values his or her contributions, the organization will reduce the amount of stress of the employee. For example, programs and processes such as flexible scheduling for employee, participatory decision making, and employee award programs, may benefit the organization by improving the job-related positivity on employees’ likelihood of reducing negative work attitude. In addition, informal support such as encouraging employees and acknowledging their hard work may also act to send the message that the organization supports them in their tasks.

## Conclusion

In summary, the present study provides evidence that perceived organizational support is correlated with job performance, with mediating effects of job satisfaction, positive affect, and affective commitment. These findings can help administrators to find ways to use POS to increase JP. Furthermore, organizational justice, including procedural and distributive justice, is an antecedent of POS and helps to improve POS in practice.

There are some limitations to this study. Firstly, it is cross-sectional design that precludes from drawing conclusions concerning the causal relationships among the observational variables. Secondly, the sample was not large, thus our power for detecting between several relationships effects was relatively low. Finally, our sample was limited to the faculty members in universities located in the north part of China which may limit the generalizability of the results. Further studies should be performed in the large-scale survey or cohort around China.

### Ethical statement

This study was approved by the ethical committee from Harbin Medical University (HMU20120314).

## Competing interests

The authors declare that they have no competing interests.

## Authors' contributions

GX and FL are responsible for the design and the manuscript prepare, TS and YL are mainly responsible for the questionnaire survey. YH and LZ are responsible for the data management and analysis. All authors read and approved the final manuscript.

## Pre-publication history

The pre-publication history for this paper can be accessed here:

http://www.biomedcentral.com/1472-6920/14/50/prepub

## Supplementary Material

Additional file 1 Demographic and Organizational Characteristics of Sample Population (n=581). Click here for file
