# Supplementary material for: The relationship between job performance and perceived organizational support in faculty members at Chinese universities: a questionnaire survey
Source: BMC Med Educ. 2014 Mar 13;14:50. doi: 10.1186/1472-6920-14-50 (PMC4008306; doi:10.1186/1472-6920-14-50)
Supplement: Additional file 1 — Demographic and Organizational Characteristics of Sample Population (n=581). [file 1472-6920-14-50-S1.doc]

**Supplementary materials**

Demographic and Organizational Characteristics of Sample Population (n=581)

| Scale | Item | N | Proportion (%) |
| --- | --- | --- | --- |
| Demographic Characteristics |  |  |  |
| Sex | Male | 220 | 37.87 |
|  | Female | 361 | 62.13 |
| Age | ≤30 | 131 | 22.55 |
|  | ~ 45 | 370 | 63.68 |
|  | ~55 | 66 | 11.36 |
|  | <55 | 14 | 2.41 |
| Educational Level | Bachelor | 124 | 21.34 |
|  | Master | 307 | 52.84 |
|  | Doctor | 150 | 25.82 |
| Tenure | <5 | 164 | 28.23 |
|  | ~10 | 212 | 36.49 |
|  | >10 | 205 | 35.28 |
| Job Title | Professor | 93 | 16.01 |
|  | Associate Professor | 146 | 25.13 |
|  | Lecture | 255 | 43.89 |
|  | Assistant Professor | 87 | 14.98 |
| Income Level | ≤4000 | 452 | 77.80 |
|  | ~ 6000 | 115 | 19.79 |
|  | >6000 | 14 | 2.41 |
| Organizational Characteristics |  |  |  |
| School Type | Directly Administered by the Ministry of Education | 14 | 2.41 |
|  | Administered by the Province | 567 | 97.59 |
| Subject Type | National Key Disciplines | 37 | 6.37 |
|  | Provincial Key Disciplines | 203 | 34.94 |
|  | Scholastic Key Disciplines | 131 | 22.55 |
|  | Others | 210 | 36.14 |
